# Diagnostic Challenges in Bone Fragility: Osteogenesis Imperfecta Case Series

**DOI:** 10.3390/biomedicines13040865

**Published:** 2025-04-03

**Authors:** Andrei Costache, Anca-Lelia Riza, Mihaela Popescu, Rebecca-Cristiana Șerban, Andreea-Mădălina Mituț-Velișcu, Ioana Streață

**Affiliations:** 1 Department of Biophysics, University of Medicine and Pharmacy of Craiova, 200349 Craiova, Romania; andrei.costache@umfcv.ro; 2 Laboratory of Human Genomics, University of Medicine and Pharmacy of Craiova, 200638 Craiova, Romania; rebecca.serban@umfcv.ro (R.-C.Ș.); ioana.streata@umfcv.ro (I.S.); 3 Regional Centre of Medical Genetics Dolj, Emergency County Hospital Craiova, 200642 Craiova, Romania; andreea.crgm@gmail.com; 4 Department of Endocrinology, University of Medicine and Pharmacy of Craiova, 200349 Craiova, Romania

**Keywords:** osteogenesis imperfecta, connective tissue disorder, genetic testing, case series

## Abstract

Osteogenesis imperfecta (OI) is a rare hereditary connective tissue disorder. Diagnosis is typically clinical; genetic testing can contribute. **Objectives**: We are presenting a case series of type I OI in Romanian patients, showcasing the difficulties in diagnostic and case management in pediatric and adult cases. **Methods**: Nine patients were referred to the Regional Centre for Medical Genetics (CRGM), Dolj, Craiova, between 2021 and 2024. Genetic testing was conducted using the commercially available kit Illumina^®^ TruSight™ One. **Results**: Most of the patients showed blue sclerae, significant fracture history, and reduced stature. In our case series, the genetic variants for seven of the cases identified are primarily in the *COL1A1* and *COL1A2* genes. Our study reveals significant clinical variability among patients, even among those with identical genetic variants. This emphasizes the importance of tailored surgical and rehabilitation programs to improve the quality of life for these patients. **Conclusions**: Our study contributes to the genetic landscape of OI. Future research should aim to include larger, more diverse cohorts and incorporate advanced genetic analysis techniques to identify additional genetic variants and mechanisms involved in OI.

## 1. Introduction

Osteogenesis imperfecta (OI) is a rare hereditary connective tissue disorder, with an estimated global incidence between 1:15,000 and 1:20,000 live births [1], and a prevalence of approximately 0.4–1.1 per 10,000 individuals [2], potentially even higher [2], as incidence and prevalence vary across regions. Often referred to as “brittle bone disease, it is the most common form of heritable bone fragility.

The phenotypic manifestations of OI are highly heterogeneous, from mild to lethal. Recurrent fractures are the main clinical feature, being often produced by minimal trauma and resulting in spinal and limb deformities [3,4]. Blue sclera, a disease hallmark, hearing loss, dental issues, and valvular disease [5,6] are frequently described. The case presentation is though polymorphic [4,7].

Diagnosis is typically clinical, supported by radiological findings such as diffuse bone demineralization, cortical thinning, and vertebral compression [7,8]. Genetic testing can confirm the diagnosis in some cases, but not all.

Defects in type I collagen, the most abundant protein in the bone extracellular matrix, are largely the underlying genetic cause [9], with collagen, type I, alpha 1—*COL1A1* and [9], collagen, type I, alpha 2—*COL1A2* genes being the most commonly involved. OI is often inherited in an autosomal dominant pattern; however, recessive inheritance and sporadic cases can occur, making family history less reliable in some instances [10,11].

OI cases vary in clinical presentation and outcome. Based on clinical, radiological, and genetic criteria, proposed classification frameworks distinguish between types, from mild (type I), with a predisposition to fractures, normal lifespan, and stature, to lethal (type II), a perinatal form, often with death shortly after birth [12]. Types III to IX are severe and moderate forms that allow survival beyond the neonatal period [13]. Type III is, for instance, a severe form with significant bone deformities and growth issues, whilst type IV is moderate with fragile bones and deformities. Distinct clinical features like hyperplastic callus formation are characteristic of type V, and mineralization defects are seen in type VI. This has been expanded to include additional types based on new genetic findings [14].

The condition is typically diagnosed during childhood; its natural history shows progression into adulthood, or it can have a late onset, making it a complex disorder to identify and manage [15].

Management of OI involves a multidisciplinary approach, including medication, orthopedic surgery, and physical therapy [16,17]. The therapeutic goal varies by phenotype. While adult and pediatric case management share the same commonalities, such as the use of bisphosphonates, in children the main objective is maintaining growth and preventing prevention, whereas in adults maintaining bone density is the focus [18]. Early intervention and comprehensive care are crucial for enhancing quality of life and minimizing complications.

Numerous case reports and case series have documented the clinical profiles of OI patients. These studies emphasize the importance of early diagnosis and management to improve patient outcomes. We are presenting a case series of type I OI in Romanian patients, showcasing the difficulties in diagnostic and case management in pediatric and adult cases.

## 2. Materials and Methods

### 2.1. Patient Inclusion and Evaluation

This study includes 9 patients evaluated for bone fragility, recurrent fractures, skeletal deformities, sclerae discoloration, and hearing impairment with onset in early adulthood in pediatric, endocrinology, or medical genetics departments and referred to the Regional Centre for Medical Genetics (CRGM), Dolj, Craiova, for genetic testing between 2022 and 2024.

Inclusion criteria for the current study were clinical OI phenotype, complete medical and familial history records, and genetic testing not available/performed. The clinical diagnosis may be based on characteristic features such as bone fragility, blue sclera, and hearing loss, among others, which are typical manifestations of OI. Exclusion criteria included previous genetic testing and inaccurate/missing family records. Clinical evaluations, including demographics, personal and familial history, clinical characteristics, the presence of non-OI diagnoses like autism spectrum disorder (ASD), ADHD, seizures, hydrocephalus, or necessity of ventriculoperitoneal shunt (VPS), imaging, and DXA studies, were obtained from referring clinicians, endocrinologists, or pediatricians.

Ethical approval for the study was granted by the local research ethics committees of the involved institutions. A written informed consent form was signed by the parents or legal guardians of the patients.

### 2.2. Genetic Testing

DNA was isolated with commercial kits from EDTA venous blood, and testing was performed using an extensive next-generation sequencing (NGS) panel, Illumina^®^ TruSight™ One on NextSeq550 IVD, MID-output to reach 100× mean coverage. Library preparation and sequencing were performed according to the manufacturer’s instructions (Illumina, San Diego, CA, USA). The gene list is available at https://www.illumina.com/content/dam/illumina-marketing/documents/products/gene_lists/gene_list_trusight_one.zip (accessed on 1 July 2024). The nf-core/sarek 2.7.1 pipeline was used to identify SNVs [19]. Mosaicism analysis was not performed for the probands or their progenitors. Validation for low coverage variants, de novo status, and/or segregation was offered with in-house capillary sequencing on a 3730xl DNA Analyzer (Life Technologies, Carlsbad, CA, USA).

The germline variants identified were annotated using the ENSEMBL variant effect predictor (VEP); online aggregate databases such as OMIM, ClinVar, and Varsome [20] were consulted. Annotated and inheritance information, where available, were used for ACMG-compliant variant classification.

We considered positive results to be the presence of pathogenic/likely pathogenic variants in genes that explained the case phenotype: (1) in a heterozygous state for dominant conditions; (2) a homozygous or compound heterozygous for recessive conditions; (3) and a hemizygous variant in an X-linked recessive gene in males. We are also discussing variants of unknown significance that may be plausible in the clinical context.

## 3. Results

### 3.1. Phenotypic Description

Most of the patients showed blue sclerae, a significant fracture history, and reduced stature. Detailed clinical data are presented in Table 1.

In our case series, significant findings such as multiple old rib fractures and severe spinal deformities were noted in the radiological assessments in seven cases, while dental issues such as dentinogenesis imperfecta were noted in two patients.

Seven patients experienced multiple fractures, while four patients reported hearing loss. All the eleven patients reported that these complications seriously impacted their ability to participate in daily activities, communication, social interactions, and overall mental well-being. Two of them required the use of hearing aids or other assistive devices, which is an additional burden for them.

The severity of clinical phenotypes significantly varied, with some patients exhibiting mild symptoms like normal stature and no fractures, while others had severe manifestations including multiple fractures, significant skeletal deformities, and hearing loss. The presence of these severe symptoms often correlated with a more challenging clinical management and a greater impact on the patient’s quality of life. The most common clinical phenotype among the patients in Table 1 is the presence of blue sclerae. This feature was observed in most of the patients. Additionally, other common phenotypes include a significant history of fractures and reduced stature. These findings highlight the variability and severity of OI in the patient cohort.

### 3.2. Genetic Findings

Of the nine individuals included in this study, the clinical diagnosis was genetically confirmed for seven cases where *COL1A1* pathogenic variants were identified (see Table 2). Genetic testing was able to confirm the clinical phenotype for 6 cases, while in 1 case a VUS was identified in the *COL1A2* gene.

*COL1A1*: c.774_785del is an in-frame variant at −20 nucleotides to the splice distance. This is a very rare variant [21]. A non-truncating, non-synonymous variant is located in a mutational hot spot and/or critical and well-established functional domain; it is currently reported as pathogenic by reputable sources such as Clinvar.

*COL1A1*: c.910del can cause a frameshift, leading to a premature stop codon and a truncated protein. Such mutations often result in severe forms of OI due to the significant disruption in collagen production [14,16,22,23,24,25,26].

Splice site mutations, like *COL1A1*: c.3369 + 2T > C, can lead to exon skipping or the use of cryptic splice sites, resulting in abnormal collagen chains. This can impair the formation of the collagen triple helix, leading to the characteristic bone fragility seen in OI [22,23,27]

*COL1A1*: c.3910C > T variant is a single-nucleotide substitution in the COL1A1 gene, leading to a glycine substitution in the collagen chain, which is critical for its triple-helix structure [22,23,28,29,30,31].

*COL1A2*: c.3641A > G is a missense variant resulting in the substitution of lysine with arginine, which may affect the collagen structure [22,23,31].

**Table 2:** Genotypic data of the case series.

Gene Variant	Variant Type	NCBI ClinVar	ACMG Score	Relevant Literature	Associated Phenotype
*COL1A1* NM_000088.3: c.774_785del p.(Ala259_Pro262del)	in frame variant, exon 11 of 51 position 24–35 of 54 (coding)	Pathogenic (*)	Pathogenic (PM1, PM4, PM2, PP5)	[21]	case2 heterozygous OI type I (OMIM#166200)
*COL1A1* NM_000088.4: c.910del p.(Arg304ValfsTer237)	frameshift variant, exon 14 of 51 position 7 of 54 (splicing, coding, NMD)	Pathogenic (*)	Pathogenic (PVS1, PM2, PP5)	[32,33]	case1 heterozygous OI type I (OMIM#166200)
*COL1A1* NM_000088.4: c.3369 + 2T > C p.?	intron 45 of 50 position 2 of 338 (splicing-ACMG, splicing, intronic)	Pathogenic (*)	Pathogenic (PVS1, PM2, PP5)	[32,33,34,35]	case3 case4 father too heterozygous OI type I (OMIM#166200)
*COL1A1* NM_000088.4: c.3910C > T p.(Gln1304Ter)	nonsense variant, exon 49 of 51 position 96 of 191 (coding, NMD)	Pathogenic (**)	Pathogenic (PVS1, PP5, PM2)	[32,33,36]	case5 case6 heterozygous OI type I (OMIM#166200)
*COL1A2* NM_000089.4: c.3641A > G p.(Lys1214Arg)	missense variant, exon 50 of 52 position 115 of 185 (coding)	-	Variant of Uncertain Significance		case7 heterozygous OI

(*) ClinVar stars.; ACMG—American College of Medical Genetics.

### 3.3. Therapeutic Intervention

In our case series, four cases with positive genetic testing for *COL1A1* pathogenic variants started therapy with bisphosphonates and reported improvement of quality of life correlated with increased bone mineral density (BMD) and reduced fracture rates.

All the OI genetic confirmed patients required orthopedic and/or surgical interventions primarily to treat fractures and correct skeletal deformities, rather than to reduce the risk of future fractures. Also, following specific physical therapy and rehabilitation programs was essential for quality-of-life improvement in these patients.

## 4. Discussion

### 4.1. Phenotype in OI

OI affects individuals globally, regardless of age, gender, or ethnicity [25]. The spectrum of clinical manifestations varies in frequency and severity between children and adults [37]; understanding the frequency of these signs and symptoms in different age groups is crucial for effective management and treatment strategies.

In children, fractures following minimal trauma have a high frequency, being the most prevalent sign, a hallmark of OI. The incidence of fractures peaks during the toddler and adolescent years; there is a decline with age in relative risk—the fracture risk is highest in younger patients and decreases as they become older, remaining the most important sign still [38]. Long bones, particularly in the lower limbs, are frequent fracture sites in children with OI, though axial skeleton fractures are also reported [39]. Hand and wrist fractures are rare [40]. Typical additional features seen, especially in children, include blue sclera, seen with a high incidence in children, especially dentinogenesis imperfecta, and possible hearing loss [37].

Approximately half of adults with OI will experience fractures and hearing loss during their lifetime [37], certainly at a higher incidence than the general population [41]. Tibia, followed by femur, are the most frequently involved fracture sites in adults with OI [42]. In adults, significant musculoskeletal symptoms such as scoliosis, respiratory insufficiency, bowel dysfunction, and chronic pain [43]. Clinical manifestations may also include pregnancy complications, cerebrovascular manifestations (valvulopathies and increased aortic diameter [44]), and vision impairment, which may arise as adults with OI age [45].

In our cohort, teeth abnormalities were not a common occurrence. Dentinogenesis imperfecta is a common dental manifestation in OI, more frequently associated with *COL1A2* mutations compared to *COL1A1*, due to the qualitative defects these mutations cause in collagen structure, a major component for bone and dentin [46]. Additionally, dental fluorosis is also a potential concern due to the altered bone metabolism in individuals with OI. Monitoring for progressive dental fluorosis or enamel defects is a must. Potential signs such as enamel discoloration, pitting, or mottling, as excessive fluoride intake often affects teeth before bones.

Quality of life questionnaires indicate pain being experienced by the vast majority of individuals, with most rating it as moderately to severely impactful. Fatigue is also prevalent, affecting more than half of adults [47].

The severity of symptoms varies, with some individuals exhibiting mild symptoms while others face severe complications [37]. While some patients may be diagnosed in childhood, others with milder forms may not be identified until adulthood, highlighting the heterogeneous nature of OI across different age groups [48].

### 4.2. Genetic Findings and Clinical Correlates

OI type I is the most common. It primarily follows autosomal dominant inheritance, most often caused by mutations in the *COL1A1* and *COL1A2* genes. Studies have consistently found a high prevalence of these mutations in OI [49].

In our case series, the genetic variants identified are primarily in the *COL1A1* and *COL1A2* genes, being consistent with those reported in other studies on OI. These genes are the most implicated in OI, and variants in these genes are known to disrupt the synthesis and structure of type I collagen, leading to the characteristic bone fragility of OI. Our findings align with the broader genetic landscape of OI, where pathogenic variants in the *COL1A1* and *COL1A2* genes are the most prevalent.

Through testing, the known genetic landscape of OI is continuously expanding: NGS can identify pathogenic variants in a significant proportion of patients, with a high diagnostic yield when clinical diagnosis is accurate [49]; WES is less used currently, though it can contribute significantly, especially in cases where targeted NGS panels do not provide conclusive results [50]; RNA sequencing of urine-derived cells has been shown to be effective in OI [51].

Genetic mutations affecting collagen synthesis and processing have been identified, such as those in the leprecan—*LEPRE*, *SERPIN*, cartilage-associated protein—*CRTAP*, and interferon-induced transmembrane protein—*IFITM* genes [22,52]—with genes being continuously discovered [50]. In some genes, autosomal recessive inheritance is possible in collagen posttranslational modification, processing, and bone mineralization, such as FK506-binding protein 10—*FKBP10*, prolyl 3-hydroxylase 1—*P3H1*, and wingless-type mmtv integration site family, member 1—*WNT1* genes [11,53]. Careful consideration for genetic counseling should be given in cases of recurrence in perinatal lethal OI due to parental mosaicism rather than recessive inheritance [10].

The large number of causative genes complicates the process of creating an optimal classification of OI. Additionally, the difficulty of creating a comprehensive classification of OI subtypes is the fact that there is no clear phenotype–genotype relationship; based on the mutation, conclusions about its clinical severity cannot readily be drawn [54]. A genotype–phenotype map can assist clinicians in correlating genetic findings with clinical manifestations, but this requires extensive genetic testing [55].

Significant intra-familial and inter-familial variability has been reported [28,56,57], particularly in dominant negative variants of the *COL1A1* gene [57]. In our cohort based on the personal history and clinical information, we also observed intrafamilial variability. In our cohort, patients carrying the same pathogenic variant exhibited mild symptoms such as normal stature and less or no fractures, while others had severe manifestations including multiple fractures or significant skeletal deformities. This variability was particularly notable in cases where the *COL1A1*: c.3369 + 2T > C variant was identified. This highlights the challenges in predicting clinical outcomes based solely on genetic findings.

To complicate things further, modifier genes [58,59] and somatic mosaicism [60] can influence the expression of OI phenotypes.

The evolving landscape of genetics means that novel variation and mechanisms are continually identified, requiring ongoing education and adaptation of counseling practices [61].

### 4.3. Diagnostic and Management Challenges in Children and Adults

Early diagnosis and multidisciplinary case management are crucial for improving medical outcomes, the quality of life, and avoiding complications [4].

Diagnosis can be complicated due to the heterogeneous clinical presentation of OI, leading to frequent misdiagnosis. In children, fractures can also be mistaken for signs of child abuse [62]. In adult patients, the symptoms of OI may be subtle or masked by other age-related conditions, e.g., reduced bone density, increased fracture risk, and joint laxity can be mistaken for more common conditions like osteoporosis or degenerative joint disease. Differential diagnosis is challenging; competing findings may not elucidate, and lack of family history may mislead [63]. In low- and middle-income countries, the lack of resources and expertise further complicates diagnosis and management, highlighting the need for increased awareness and education [64].

Clinical features like blue sclera, dentinogenesis imperfecta, and hearing loss can aid in diagnosis, but these are not always present; mild and moderate forms of OI often lack distinct clinical criteria, making them difficult to diagnose based solely on clinical features [48,55].

Imaging techniques such as ultrasound, radiography, CT, and MRI are essential for diagnosing OI, especially in differentiating it from non-accidental trauma in children [48]. Conventional radiography is pivotal in identifying the hallmark features of OI, such as generalized osteopenia or osteoporosis, bone deformities, and multiple fractures of varying ages [65]. Dual-energy X-Ray Absorptiometry (DXA) is widely used to measure areal Bone Mineral Density (aBMD), although it has limitations in fully characterizing bone fragility in OI [65]. In prenatal settings, ultrasound can detect skeletal hypomineralization and bone deformities, particularly in severe OI types. In milder forms like OI type I, subtle signs such as increased nuchal translucency and thin skull bones may be observed [66].

Genetic testing is crucial for diagnosing and understanding this genetically heterogeneous disorder, but the genetic confirmation may be absent [4]—then the diagnosis mainly relies on the clinical presentation. Nonetheless, genetic testing remains a valuable tool. Genetic counseling is an indispensable part of managing OI, providing the patient and their family with essential information to help navigate the complexities of inheritance, prenatal diagnosis, and family planning, and supports personalized treatment plans while offering an otherwise overlooked but significant component of psychosocial support [67,68,69].

OI management requires a team of specialists, including pediatricians, geneticists, endocrinologists, orthopedic surgeons, physiotherapists, and nutritionists, to address the diverse needs of patients [16,26].

Current management strategies focus on minimizing fracture rates and managing deformities [16] and include pharmacological treatments, surgical interventions, nutritional support, and emerging therapies.

Bisphosphonates are the cornerstone of medical treatment, shown to increase bone mineral density (BMD) and reduce fracture rates, although their long-term effects on fracture risk remain uncertain [26,70].

Newer therapies, such as teriparatide, denosumab, and sclerostin inhibitors, have shown promise in increasing BMD but require further research to confirm their efficacy in reducing fractures [26,71]. Emerging therapies such as anti-RANKL antibodies, sclerostin inhibitors, recombinant human parathormone, and TGF-β, which have shown promising results in various studies [71]. Gene therapy and stem cell transplantation are promising areas of research, aiming to address the underlying genetic defects in OI, as are CRISPR-Cas9 gene editing techniques [72]. Enhanced research methodologies and larger, diverse studies are necessary to improve the generalizability of findings and optimize patient care.

Orthopedic surgery is crucial for fracture fixation and correcting limb deformities, while physical therapy and rehabilitation are essential for improving mobility and functional capacity [73]. Surgical interventions are aimed at reducing fracture risk and improving quality of life [74], but treatment responses may vary based on the type of OI [75].

Individualized nutritional support is critical, as many patients with OI do not meet recommended calcium and vitamin D intake levels, which are vital for bone health [76].

OI in children can lead to reduced height and lean mass, with a relatively higher fat mass, complicating nutritional assessments and interventions [77]. Gait deviations are common due to skeletal deformities, impacting mobility and requiring tailored physical therapy [78].

OI children and adults often experience a lower quality of life compared to their peers, with significant challenges in physical, emotional, and social functioning [79], which also need to be addressed.

### 4.4. Limitations and Perspectives

Our case series includes a limited number of patients, which may not be representative of the broader population with OI. Future studies should aim to include a larger and more diverse cohort of patients. This would improve the applicability of our findings and provide a more comprehensive understanding of the clinical and genetic variability of OI in the Romanian population.

It remains challenging to capture the full spectrum of OI presentation, treatment requirements, and the socioeconomic impact. National registries play a crucial role in covering significant gaps in understanding the incidence, prevalence, clinical characteristics, and management of OI [80]. Valuable data that can inform healthcare policies and improve long-term patient care. Examples of lessons learned were the better understanding of fracture characteristics during the entire life span [38], the need to focus on the incidence of cataracts and glaucoma, or osteoarthritis [81]. Natural history initiatives [39] need to be completed by genotype–phenotype correlations [53] and better understanding of socio-economic impact [2]. Such initiatives can only lead to increased awareness and improved guidelines for managing OI-related complications [82]. Also, conducting long-term follow-up studies would be beneficial to understand the progression of OI and the long-term outcomes of various treatments. This would provide valuable insights into the natural history of the disease and help in developing more effective management strategies.

We have discussed current management strategies, all of which require coordination to reach optimal patient outcomes. Though, standardized guidelines do not currently exist. Unquestionably needed, developing comprehensive guidelines should address the clinical and genetic heterogeneity, the diversity of treatment choices, and the importance of the multidisciplinary approach to care. Such guidelines would ensure consistent and effective care across different healthcare settings [83] and would facilitate the integration of new therapies into clinical practice as well as help in the design of clinical trials to close current gaps [26,71].

As we have seen in our cohort, while genetic testing was performed, it was not able identify a genetic cause for the otherwise suggestive clinical phenotype in all cases. Additionally, mosaicism analysis was not performed, which could have provided more comprehensive insights into the genetic underpinnings of OI. Incorporating more advanced genetic analysis techniques, such as whole-exome sequencing (WES) and RNA sequencing, could help identify additional genetic variants and mechanisms involved in OI. This would contribute to a better understanding of the genetic basis of the disease and potentially lead to the discovery of novel therapeutic targets.

## 5. Conclusions

Understanding this heterogeneous rare connective tissue disorder characterized by fragile bones due to defects mainly in type 1 collagen leads to a range of clinical manifestations, including frequent fractures, bone deformities, and various extra-skeletal symptoms.

The study highlights the genetic heterogeneity of OI, with pathogenic variants identified primarily in the *COL1A1* and *COL1A2* genes. These findings align with the broader genetic landscape of OI, where pathogenic variants in these genes are the most prevalent.

Our study reveals significant clinical variability among patients, even those with the same genetic variant. This variability complicates the prediction of clinical outcomes based solely on genetic findings and underscores the need for personalized management strategies.

All genetically confirmed OI patients from our study required orthopedic and/or surgical interventions primarily to treat fractures and correct skeletal deformities, rather than to reduce the risk of future fractures. This emphasizes the importance of tailored surgical and rehabilitation programs to improve the quality of life for these patients.

The study acknowledges limitations such as the small sample size and lack of long-term follow-up data. Despite these, the study contributes to increasing the knowledge regarding the genetic landscape of OI in Romania. Future research should aim to include larger, more diverse cohorts and incorporate advanced genetic analysis techniques to identify additional genetic variants and mechanisms involved in OI.

## Figures and Tables

**Table 1:** Phenotypic data of the case series.

	case1	case2	case3	case4	case5	case6	case7	case8	case9
Onset	fracture at 4 years old	blue sclerae at 2 years old	fracture at 12 years old	fracture at 13 years old		ankle fracture at birth		congenital bilateral hip luxation	
Age of genetic diagnosis	7 years old	2 years	22 years old	17 years old	67 years old	47 years old	64 years old	4 years old	
Stature	normal	reduced	normal	normal	reduced	reduced	reduced	reduced	reduced
Fractures	multiple	none	multiple	multiple	multiple	multiple	multiple	multiple	none
Skeletal									
Bone deformity	no	yes	no	no	yes	yes	yes	yes	yes
Teeth	dental anomalies	no	no	no	no	no	dentinogenesis imperfecta	no	no
Skull	normal	normal	normal	normal	normal	normal	normal	normal	normal
Spine	normal	normal	normal	thoraco-lumbar kyphoscoliosis	dextroconvex thoracic kyphoscoliosis	s-shaped lumbar scoliosis	thoraco-lumbar kyphoscoliosis	thoraco-lumbar scoliosis	double thoracic and lumbar dextroconvex scoliosis
Pelvis	normal	normal	normal	normal	normal	normal	normal	normal	normal
Limbs	multiple fractures	no	multiple fractures	multiple fractures	multiple fractures	multiple fractures	multiple fractures	multiple fractures	none
Blue sclerae	yes	yes	yes	yes	yes	yes	yes	dark colored	yes
Hearing loss	no	no	no	no	yes	yes	yes	no	yes
Skin	normal	normal	normal	normal	bruising	normal	normal	bruising	normal
Cardiovascular	normal	normal	normal	normal	hipertensive	normal	hipertensive	normal	hipertensive
Radiology/DXA	z-score 1.4 sd at 5 years	none	none	lumbar spine z-score (−3 sd)	chest X-Ray presenting signs of multiple old rib fractures	lumbar spine t-score (−4.9 sd)	lumbar spine t-score (−6.4 sd), femoral neck t score (−3.3 sd)	spine X-Ray: thoraco-lumbar dextroconvex scoliosis with cobb angle of 40 degrees	lumbar spine t-score (−3 sd)
Miscellaneous			sibling of case4	sibling of case3	mother of case6	daughter of case5		congenital hiatal hernia	
Family history		father with cardiac anomalies	father and sibling positive for OI	father and sibling positive for OI	daughter positive for OI	mother positive for OI			

sd—standard deviation.

## Data Availability

Data are contained within the article.
